# COVID-19 Prediction Using Black-Box Based Pearson Correlation Approach

**DOI:** 10.3390/diagnostics13071264

**Published:** 2023-03-27

**Authors:** Dilber Uzun Ozsahin, Efe Precious Onakpojeruo, Basil Bartholomew Duwa, Abdullahi Garba Usman, Sani Isah Abba, Berna Uzun

**Affiliations:** 1 Department of Medical Diagnostic Imaging, College of Health Science, University of Sharjah, Sharjah 27272, United Arab Emirates; 2 Operational Research Centre in Healthcare, Near East University, TRNC Mersin 10, Nicosia 99138, Turkey; 3 Department of Analytical Chemistry, Faculty of Pharmacy, Near East University, TRNC Mersin 10, Nicosia 99138, Turkey; 4 Interdisciplinary Research Center for Membranes and Water Security, King Fahd University of Petroleum & Minerals, Dhahran 31261, Saudi Arabia; 5 Department of Statistics, Carlos III University of Madrid, 28903 Madrid, Spain; 6 Department of Mathematics, Near East University, TRNC Mersin 10, Nicosia 99138, Turkey

**Keywords:** coronavirus, MLR, Israel, COVID-19, ANN

## Abstract

The novel coronavirus (COVID-19), also known as SARS-CoV-2, is a highly contagious respiratory disease that first emerged in Wuhan, China in 2019 and has since become a global pandemic. The virus is spread through respiratory droplets produced when an infected person coughs or sneezes, and it can lead to a range of symptoms, from mild to severe. Some people may not have any symptoms at all and can still spread the virus to others. The best way to prevent the spread of COVID-19 is to practice good hygiene. It is also important to follow the guidelines set by local health authorities, such as physical distancing and quarantine measures. The World Health Organization (WHO), on the other hand, has classified this virus as a pandemic, and as a result, all nations are attempting to exert control and secure all public spaces. The current study aimed to (I) compare the weekly COVID-19 cases between Israel and Greece, (II) compare the monthly COVID-19 mortality cases between Israel and Greece, (III) evaluate and report the influence of the vaccination rate on COVID-19 mortality cases in Israel, and (IV) predict the number of COVID-19 cases in Israel. The advantage of completing these tasks is the minimization of the spread of the virus by deploying different mitigations. To attain our objective, a correlation analysis was carried out, and two distinct artificial intelligence (AI)-based models—specifically, an artificial neural network (ANN) and a classical multiple linear regression (MLR)—were developed for the prediction of COVID-19 cases in Greece and Israel by utilizing related variables as the input variables for the models. For the evaluation of the models, four evaluation metrics (determination coefficient (R2), mean square error (MSE), root mean square error (RMSE), and correlation coefficient (R)) were considered in order to determine the performance of the deployed models. From a variety of perspectives, the corresponding determination coefficient (R2) demonstrated the statistical advantages of MLR over the ANN model by following a linear pattern. The MLR predictive model was both efficient and accurate, with 98% accuracy, while ANN showed 94% accuracy in the effective prediction of COVID-19 cases.

## 1. Introduction

In December 2019, Wuhan, China reported the first cases of COVID-19, also known as the novel coronavirus or SARS-CoV-2. The virus is thought to have originated in bats and was transmitted to humans through an intermediate animal host, possibly pangolins. The first cases of COVID-19 were identified in Wuhan in December 2019 and were initially linked to a seafood market in the city [1]. However, it was later determined that the virus was also present in other areas of the market, suggesting that it was being transmitted from person to person. As the number of cases in Wuhan increased, the Chinese government implemented measures to try to control the spread of the virus, including quarantining affected areas and suspending travel to and from Wuhan. However, the virus had already spread to other parts of China and other countries, and it quickly became a global pandemic. Since the outbreak began, there have been more than 100 million confirmed cases of COVID-19 and more than 2 million deaths worldwide. The pandemic has had a significant impact on global health, economies, and daily life, with many countries implementing measures, such as lockdowns, travel restrictions, and mask mandates, in an effort to slow the spread of the virus [1,2]. The COVID-19 pandemic has affected countries all around the world, with some regions being more severely impacted than others. As of January 2021, the countries with the highest number of confirmed cases of COVID-19 included the United States, India, and Brazil. These countries have also reported high numbers of deaths due to the virus. Other countries that have been significantly affected by the pandemic include Russia, Argentina, Mexico, and Colombia. It is important to note that the impact of the pandemic can vary within countries as well. Some regions or populations may be more severely affected due to a variety of factors, such as the availability of medical resources and the effectiveness of containment measures. It is also worth noting that the reported number of cases and deaths can be affected by a variety of factors, such as the availability of testing and the accuracy of reporting. As a result, it is possible that the true impact of the pandemic may be different from what has been reported [3].

SARS-CoV-2 is primarily spread through respiratory droplets produced when an infected person talks, coughs, or sneezes. These droplets can be inhaled by people who are in close proximity to the infected person. This is why it is important to practice good hygiene. It is important to note that COVID-19 can be transmitted by people who do not have any symptoms, so it is important to follow guidelines set by health authorities [4].

The symptoms of COVID-19 can range from mild to severe. The most common symptoms of COVID-19 include fever, dry cough, and tiredness. The less common symptoms include aches/pains, sore throat, diarrhea, conjunctivitis, headache, loss of taste or smell, rash on the skin, and discoloration of the fingers or toes. Severe symptoms, which may require hospitalization, include difficulty breathing or shortness of breath, chest pain or pressure, and loss of speech or movement [4].

The mortality rate of COVID-19 can vary depending on a number of factors, including the age and overall health of the individual, the severity of the illness, and the availability of medical treatment. Overall, the mortality rate for COVID-19 is thought to be around 2%, although this number can vary widely depending on the population being studied. For example, the mortality rate may be higher among older individuals and those with underlying health conditions, such as heart disease or diabetes. It is important to note that the mortality rate can also be affected by the availability of medical resources and the effectiveness of interventions, such as oxygen therapy and mechanical ventilation. In situations where these interventions are not available or are not used in a timely manner, the mortality rate may be higher. It is also important to note that the true mortality rate of COVID-19 may be higher than reported due to underreporting and the fact that some people who have the virus may not have been tested or may not have had their cases reported [5].

There are a number of different tests that can be used to diagnose COVID-19. The type of test used can depend on a number of factors, including the availability of the test, the severity of the illness, and the stage of the infection. The most common types of tests for COVID-19 include molecular tests, which detect the genetic material of the virus in a sample from the respiratory tract (such as a swab from the nose or throat); antigen tests, which detect proteins from the virus in a sample from the respiratory tract; and antibody tests, which detect antibodies produced by the body in response to the virus in a blood sample. Molecular tests, also known as PCR (polymerase chain reaction) tests, are the most accurate and are typically used to diagnose active infections. Antigen tests are generally less accurate but can provide results more quickly. Antibody tests can be used to detect past infections, but they are not as reliable for diagnosing active infections. It is important to note that the accuracy and availability of these tests can vary [6].

It is crucial to compare and contrast the rates of positive cases, the number of recoveries, the comparison of mortality cases, evaluate the effects of the vaccines, and examine other factors affecting the spread of this virus due to a lack of test kits, ventilators, oxygen tanks, hospital beds, and proper treatment or vaccine. In a similar vein, adequate preparations can be made to reduce casualties and improve situational awareness [7]. For instance, the government can prepare for the expected number of cases up until a certain day by analyzing the data in this study and deciding, in advance, what kind of medical supplies are needed or what kind of precautions can be taken to reduce the number of casualties.

Recently, machine learning techniques have increasingly been used in the healthcare sector, especially for the quick and precise prediction of COVID-19 infection. A study by [8] reported predicated cases of COVID-19 using the MLR model. Another study by [9] predicted the spread of COVID-19 using a machine-learning model called the support vector regression method. When they were evaluated using the evaluation parameters, the models showed efficiency and accuracy in predicting COVID-19 cases. Similarly, a study by [10] was able to identify factors that are associated with the transmission of COVID-19 using the machine learning approach. Another study by [11] used the least square support vector machine models to predict COVID-19 confirmed cases. DNA sequences based on machine learning were deployed to identify the biomarkers of COVID-19 in one prior study [12]. A short-term prediction of COVID-19 cases in Brazil was reported in another study [13]. A review by [14] reported the efficiency of artificial intelligence models in forecasting and diagnosing COVID-19. Similarly, review studies [1,15,16] have reported the diagnosis, classification, and prediction of COVID-19 from chest CT images using artificial intelligence models. Further, according to a study analyzing the effect of environmental parameters on forecasting daily COVID-19 cases, the inclusion of temperature and relative humidity as additional inputs in a multivariate LSTM model resulted in an average of 64% improvement in performance compared to univariate models. The study used data from 9 cities across India, the USA, and Sweden with varying climatic zones and found that correlations with temperature were generally positive for cold regions and negative for warm regions, while relative humidity showed mixed correlations. The results suggest that the inclusion of environmental parameters could aid in improving the management and preparedness of the healthcare system during the pandemic, although other confounding factors can affect the forecasting power [17]. Similarly, a novel multi-stage deep learning model has been presented to forecast the number of COVID-19 cases and deaths for each US state at a weekly level for a forecast horizon of 1–4 weeks. The model relies on epidemiological, mobility, survey, climate, demographic, and SARS-CoV-2 variant frequencies data and has been shown to consistently outperform the CDC ensemble model for all evaluation metrics in multiple spatiotemporal settings, especially for the longer-term forecast horizon. The study highlights the potential value of variant frequency data for use in short-term forecasting to identify forthcoming surges driven by new variants. The proposed forecasting framework improves upon the available state-of-the-art forecasting tools currently used to support public health decision-making with respect to COVID-19 risk [18]. Finally, a study by [19] aimed to predict the incidence of COVID-19 in Iran using data obtained from the Google Trends website. Linear regression and LSTM models were used, and the most effective factors aside from the previous day’s incidence were the search frequency of handwashing, hand sanitizer, and antiseptic topics. The results suggested that data mining algorithms can be employed to predict trends of outbreaks and support policymakers and healthcare managers in planning and allocating healthcare resources accordingly.

Based on what has been presented in our reviewed studies so far, it is clear that most studies employing data-driven models applied classical linear models, such as MLR and others like it, but they also made use of traditional non-linear models (e.g., SVM, LSTM, etc.). To the best of the authors’ knowledge, however, since the announcement of AI-based models in the field of health sciences, no article has been published depicting a black-box-based Pearson correlation approach combining the applications of ANN and the traditional linear regression MLR for the prediction of COVID-19 cases with a focus in Israel and Greece. This would be a significant advance in the understanding of the COVID-19 pandemic. However, this is the case despite the fact that ANN and MLR are two of the most popular statistical approaches. This study had four objectives. The first was to compare and contrast the weekly COVID-19 cases in different countries, such as Israel and Greece. The second goal was to analyze the differences between Israel and Greece in terms of monthly COVID-19 mortality cases. Thirdly, we aimed to determine the correlation between Israel’s vaccination rate and the number of deaths caused by COVID-19 and to report the findings. Lastly, we aimed to predict the incidence of COVID-19 cases in Israel. The benefit of completing these tasks is that various mitigations can be put into place, reducing the likelihood that the virus will spread. We used a correlation analysis and two different AI-based models, an ANN and a classical linear regression MLR, to predict cases of COVID-19 in Israel by using correlated variables as inputs. To learn how well each model performed in practice, we calculated its determination coefficient (R^2^), mean squared error (MSE), root mean squared error (RMSE), and correlation coefficient (R). The promising outcomes showed the superiority of the MLR predictive model, in terms of efficiency and accuracy, in the effective prediction of future COVID-19 cases.

## 2. Material and Methods

### 2.1. Data Collection

The COVID-19 cases dataset was collected from kaggle.com and represents cases from all continents. There was a total of 231,871 COVID-19 case records in the database, along with 53 attributes pertaining to those cases in various parts of the world. The experimental dataset was comprised of observations made from 2020 to 2022. In order to train the proposed model, a dataset with 52 input variables was used.

### 2.2. Filtering and Pre-Processing the Data

During this process, unnecessary columns were eliminated, and missing values were added [20,21]. The next step was to arrange the dataset according to the order that would enable evaluation. During the pre-processing phase [22], a table of records was converted into a more usable format through a series of steps:

- Data from two countries (Greece and Israel) were collected from the overall dataset to enable us to carry out the evaluation.
- Columns and rows containing no valid data were deleted.
- For our prediction, only datasets from Israel were used to train the model.
- Data normalization [21,22] was carried out prior to modeling using Equation (1).

(1) $$y=0.05+\left(0.95\times \left(\frac{x-x_{\min}}{x_{\max}-x_{\min}}\right)\right)$$

where x is the measured data $x_{\min}$ and $x_{\max}$ are the minimum and maximum values, respectively.

### 2.3. Prediction Models

#### 2.3.1. Artificial Neural Network (ANN)

Machine learning algorithms that mimic the human brain in structure and operation are known as artificial neural networks. They process and transmit data via layers of “neurons” (cells) that are connected to one another. A neuron’s activation is the result of a simple computation that the algorithm performs based on the information it receives from other neurons. The results of this calculation are then communicated to the neurons of the following layer. With some tweaks to the weights and biases of the connections between neurons, an artificial neural network can learn to perform a wide variety of tasks. Image recognition, text translation, and stock market forecasting are just some of the many tasks that can be taught to a neural network [23].

Feedforward neural networks, convolutional neural networks, and recurrent neural networks are just a few examples of the many varieties of artificial neural networks. Each neural network is built to accomplish a specific task, and this determines its unique structure. To perform a given task, an artificial neural network’s formula will vary depending on the type of network used, although neural networks frequently employ a small set of standard mathematical operations. The dot product, which quantifies the degree to which two vectors are similar, is one of the most fundamental operations in neural networks. The formula for the dot product of two vectors, x and w, is as follows:

dot (x, w) = ∑x[i] * w[i] (2)

where x[i] and w[i] are the i-th elements of the vectors x and w, respectively, and the sum is taken over all elements of the vectors.

Another common operation used in neural networks is the activation function, which is applied to the output of the dot product to determine the output of a neuron. There are many different activation functions that can be used, such as the sigmoid function, the tanh function, and the ReLU function. The specific formula for an activation function will depend on the function being used. For example, the sigmoid function is defined as:

f(x) = 1/(1 + e^−x^) (3)

where e is the base of the natural logarithm.

Finally, a loss function, which evaluates how far the neural network’s prediction deviates from the actual result, is typically used to compute the neural network’s output. Adjusting the neural network’s weights and biases to minimize the loss is how it is optimized. The neural network’s loss function formula is unique to the task at hand.

For many challenging problems in science and technology, ANNs trained with FFNN-BP have proven to be invaluable tools.

Additionally, FFNN-BP calls for training the network with trained input data, which is then processed within the network and transmitted to the output layer. If mistakes are made, they are passed around the system until the desired result is achieved. The FFNN-BP algorithm’s central idea is to minimize the network’s error so that it can fully understand the training data and make more precise predictions of the true value [22]. During operation, the initial weights are multiplied by the inputs, and the resulting value is transferred to the second layer, where it remains until it reaches the output layer, as shown in the following equation:

(4) $$z_{i}=\sum_{j=1}^{m}w_{ij}x_{ij}$$

where *x_ij_* is an illustration of the input, *y_i_* is the consequent sum of outputs from the ith node, and zi is the weight transferred from the *j*th input to the *i*th node. Error is calculated by subtracting the predicted values from the goal value, and this is what backpropagation is utilized for. In most cases, the output layer is used as a starting point, followed by the input layer. The error node, j, in layer l is represented by the symbol (l)_j_, which indicates the discrepancy.

The mathematical expression for the error term for a training set (x_j_, y_j_) can be found below in Equation form:

(5) $$e_{p\;}=y_{d}-y_{a}\;\;\;\;\;\;\;\;\;$$

if $y_{d}$ represents the output of neuron p and $y_{a}$ represents the actual output produced by the training model.

**Figure 1:**
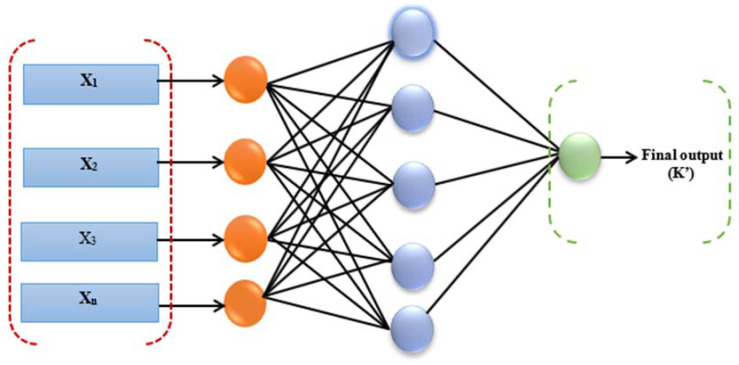
The three-layer feed-forward neural network architecture used in the current study.

However, the generalization ability and capacity of the neural network can be impacted by the presence of a large number of neurons in the hidden layer. Because lower neurons are unable to generate the required level of prediction accuracy, this raises the computational burden. One way to think about learning is as an ongoing process in which the biases and connection weights are tweaked until the desired output is achieved. This process of fine-tuning will keep going on until the desired result is achieved. This process may be performed under close observation or independently. Reducing the dispersion between the computed value and the desired value is a common supervised learning objective. Figure 1, demonstrates the three-layer, feed-forward neural network architecture used in the current study.

#### 2.3.2. Multiple Linear Regression (MLR)

Modeling the linear relationship between a dependent variable and a set of independent variables is the goal of MLR, a statistical technique. A dependent variable’s value can be predicted given the values of the independent variables [24].

The dependent variable in an MLR model is modeled as a linear combination of the independent variables plus an error term that is assumed to be random. Model parameters, or the coefficients of the independent variables, are estimated with the help of an optimization algorithm, such as least squares.

The general form of an MLR model can be written as:

y = b_0_ + b_1_x_1_ + b_2_x_2_ + ... + b_n_ * x_n_ + e (6)

where y is the dependent variable, x_1_, x_2_, ..., x_n_ are the independent variables, b_0_, b_1_, ..., b_n_ are the model parameters, and e is the random error term.

MLR is widely used in many fields, including economics, finance, and engineering, to analyze and predict the relationships between variables. It is a simple and effective method for modeling linear relationships, but it may not be suitable for modeling nonlinear relationships.

### 2.4. Model Validation

The primary focus of data-driven models is to obtain reliable forecasts for undiscovered datasets by fitting the model to the available data in accordance with the indicators being used [25]. In most cases, this is achieved by adjusting the model to better suit the data. Overfitting creates situations where training success does not necessarily translate to test success [26]. For this reason, overfitting is problematic. Holdout, leave-one-out, k-fold cross-validation, and other validation methods are just some of the options available. Cross-validation, also known as k-fold cross-validation, is one such method. As an alternative to the complex k-fold method, the holdout strategy is often viewed as more user-friendly [27]. At this point, the data are typically split randomly in half, with one half used for training and the other for testing [28]. One of the main advantages of the k-fold cross-validation mechanism is that in each round, the validation set and the training sets are completely separate from one another. As a result, a performance goal is defined, which serves as a cornerstone for subsequent model optimization. Considering the 4-fold cross-validation, we divide the collected data into two samples, with 70% going toward the training phase and 30% to the testing phase. It’s worth noting that there are different approaches that can be taken to validate and divide the data [29,30].

### 2.5. Model Performance Criteria

In order to determine how well a data-driven method performed, it is necessary to compare the predicted values with the actual ones that were collected [31]. The models were evaluated in this study using several different statistical error measures, as well as the determination coefficient (R2) as a goodness-of-fit measure [32]. Other measures used included the mean squared error (MSE), the root mean squared error (RMSE), the mean absolute percentage error (MAPE), and the correlation coefficient (R):

(7) $$R^{2}=1-\frac{\sum_{j=1}^{N}{\left[{\left(Y\right)}_{obs,j}-{\left(Y\right)}_{com,j}\right]}^{2}}{\sum_{j=1}^{N}{\left[{\left(Y\right)}_{obs,j}-{\bar{\left(Y\right)}}_{obs,j}\right]}^{2}}.\;$$

(8) $$RMSE=\sqrt{\frac{\sum_{i=1}^{N}{\left(Y_{obsi}-Y_{comi}\right)}^{2}}{N}}$$

(9) $$\mathrm{MSE}=\frac{1}{N}\;\sum_{i=1}^{N}{\left(Y_{obsi}-Y_{comi}\right)}^{2}$$

(10) $$R=\frac{\sum_{i=1}^{N}\left(Y_{obs}-{\bar{Y}}_{obs}\right)\left(Y_{com}-{\bar{Y}}_{com}\right)}{\sqrt{\sum_{i=1}^{N}{\left(Y_{obs}-{\bar{Y}}_{obs}\right)}^{2}}\sum_{i=1}^{N}{\left(Y_{com}-{\bar{Y}}_{com}\right)}^{2}}$$

where *N* is the number of data points, *Y* obsi is the number of data points that have been observed, Y is the average value of the observed data, and *Y* comi is the computed value.

## 3. Application of Results and Discussion

Data-driven methods, such as MLR and ANN, were used to predict COVID-19 cases in Israel based on related independent variables. Prior to detailing the model calibration, the results of the statistical analysis of the data have been presented in Table 1. Analyzing data helps determine the navigational and scientific value of the data, thus fixing problems that could otherwise prevent an accurate simulation of the results. MATLAB 9.3 (R2019A) was used in the process of developing the model that was used in the construction of the ANN model. To predict cases of COVID-19 in Israel, R-programming software 2017 and Excel were used to run correlation analyses. To develop the classical linear regression (MLR) model using Excel, the average of the segmented, data-driven correlations of 53 input variables was taken.

### 3.1. Descriptive Analysis

**Figure 2:**
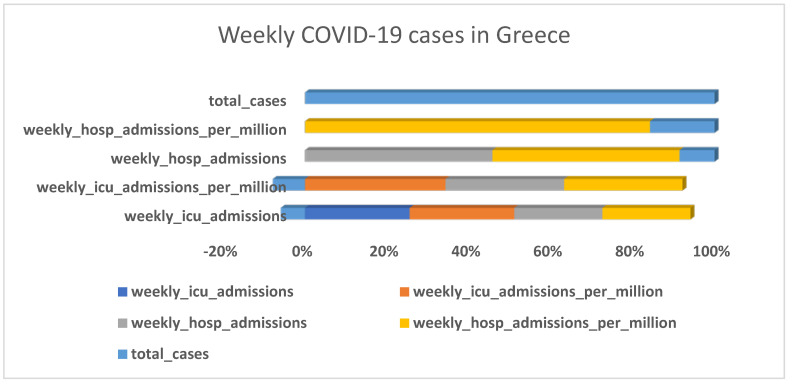
Weekly COVID-19 cases in Greece.

**Figure 3:**
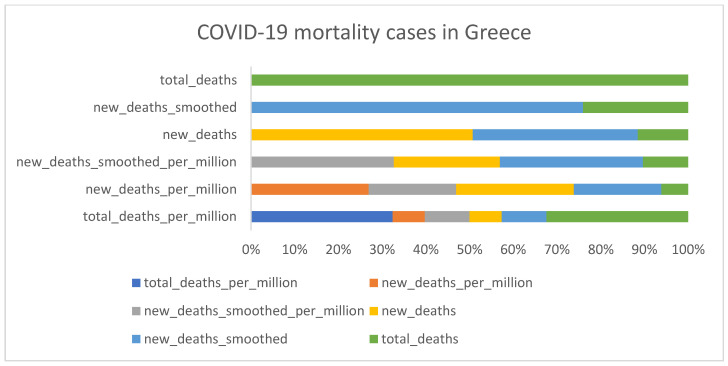
COVID-19 mortality cases in Greece.

**Figure 4:**
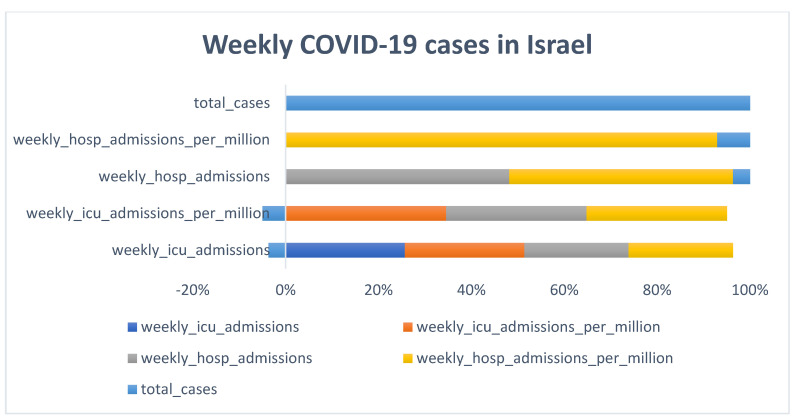
Weekly COVID-19 cases in Israel.

**Figure 5:**
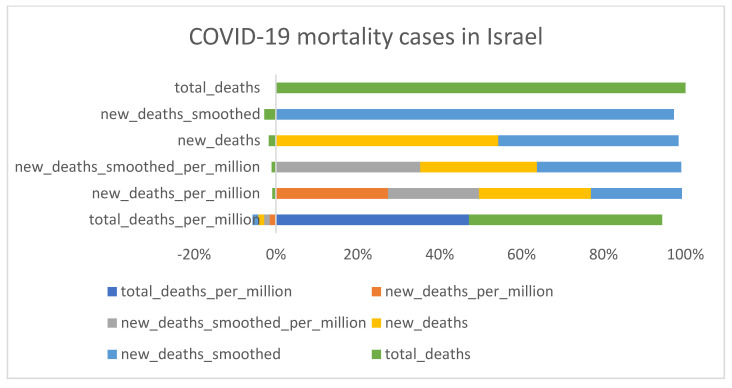
COVID-19 mortality cases in Israel.

Figure 2, Figure 3, Figure 4 and Figure 5 shows that the numbers of reported cases of COVID-19 in Greece and Israel were significantly correlated with the number of patients admitted to hospitals each week. On the other hand, when compared to Greece, Israel reported a greater number of cases of COVID-19 each week. In addition to this, Greece reported an overall increase in the number of deaths as well as a regular increase in the number of newly reported deaths on a monthly basis, as shown in Figure 2, Figure 3, Figure 4 and Figure 5. There was not even a hint of an inverse correlation found when looking at the input variables, which, as shown in Figure 2, Figure 3, Figure 4 and Figure 5, all contributed to an increase in the number of cases of COVID-19 and the accumulated death cases for Greece and Israel, respectively.

**Figure 6:**
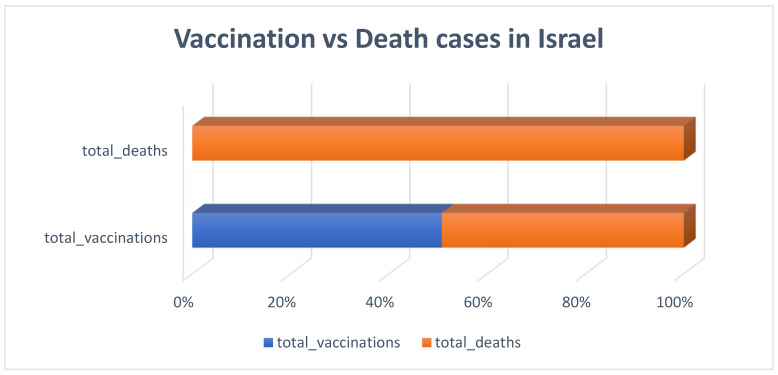
Vaccination influence on COVID-19 mortality cases in Israel.

As demonstrated in Figure 6, there was a strong correlation between total vaccinations and total death cases in Israel. The rate of vaccination, therefore, did not influence the mortality rate.

### 3.2. Results of the Models

**Figure 7:**
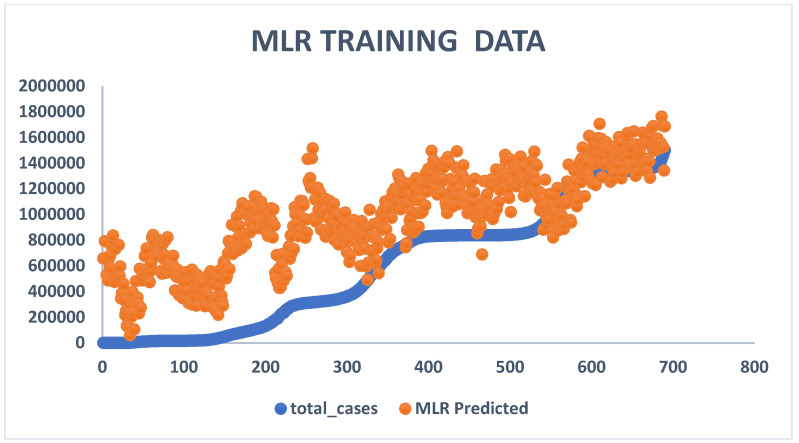
MLR training data showing experimental and predicted values.

**Figure 8:**
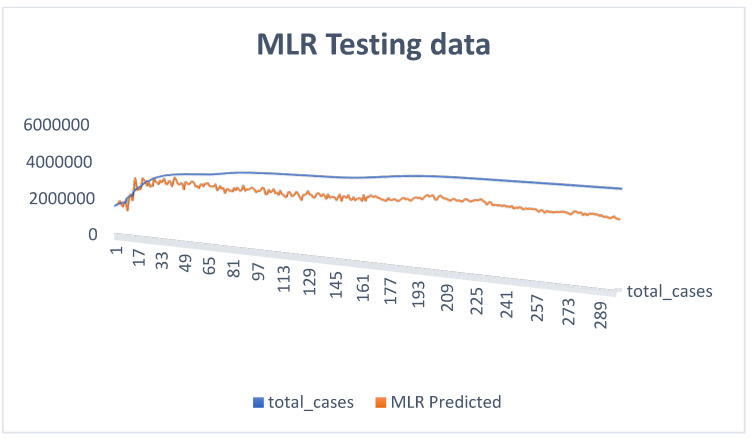
MLR testing data showing experimental and predicted values.

**Figure 9:**
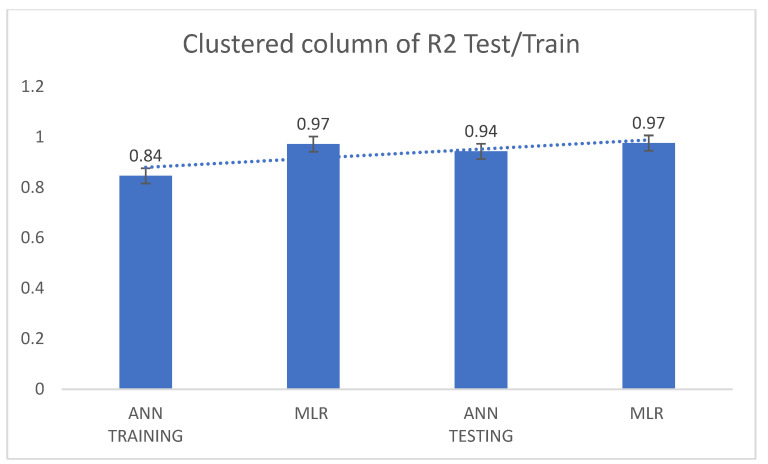
Clustered column of the determination coefficients (R^2^) of the models in both the training and testing stages.

**Figure 10:**
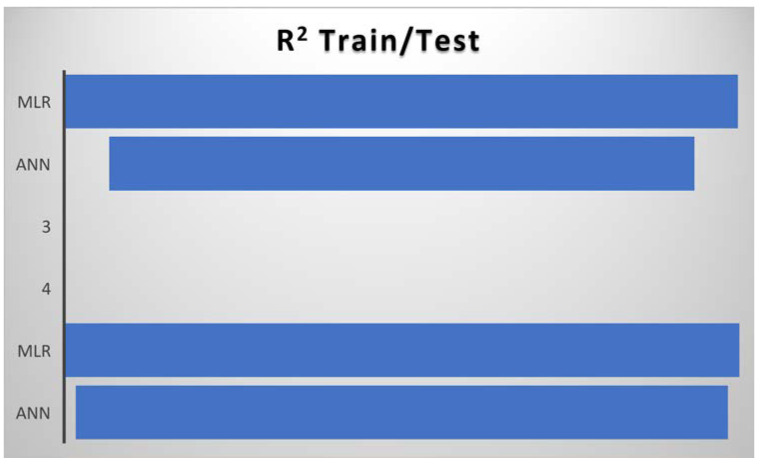
Funnel chart of determination coefficients (R^2^) of the models in both the training and testing stages.

From the comparative predictive results of the models, as seen in Table 1, it can be clearly observed that the MLR model and the ANN model were capable of predicting COVID-19 cases. Therefore, the MLR and ANN models can act as reliable tools in predicting COVID-19 cases in the future. The results in Table 1 can be further discussed comparatively based on their corresponding determination coefficients (R^2^) using a clustered column and funnel chart (see Figure 7, Figure 8, Figure 9 and Figure 10). For R^2^ and R, the training results for ANN were 84% and 91%, while the results for MLR were 97% and 98% accuracy. Further, for the testing results, R^2^ and R for ANN were 94% and 97%, while the results for MLR were 97% and 98% accuracy. We can present and organize the findings from our predictive comparison in the following way: Regarding the prediction of COVID-19, MLR was superior to ANN, and this result is similar to the findings of [6,7,23,24,33,34]. Additionally, ref. [35] showed that the ANN model adopted to estimate and quantify the impact of the response measures imposed by many countries around the world to suppress the rapid spread of the COVID-19 pandemic on urban traffic mobility was capable of mapping the complex relationship between traffic flows and the response measures with a high level of accuracy and good performance. The predicted values were close to the observed ones, with a coefficient of determination (R^2^) of 0.9761. Similarly, a study by [36] adopted the ANN model to forecast the number of daily cases and deaths caused by COVID-19, in a generalized way, to fit different countries’ spread. The ANN model developed in this study showed 86% overall accuracy in predicting the mortality rate and 87% in predicting the number of cases, which makes it a reliable tool to predict the spread of the virus. Finally, a study by [37] predicted the daily COVID-19 cases in 10 African countries using machine learning models. The study concludes that ANN was among the models that offered accurate predictions that could assist governments and health organizations in making informed decisions and evaluating measures to prevent and control COVID-19.

The MLR model was found to be a satisfactory and reliable tool based on the comparative outcome. Moreover, the corresponding determination coefficients (R^2^) in Table 1 demonstrate the statistical advantages of MLR over the ANN model, i.e., the data follows a linear pattern. Additionally, the ANN model produces negative values during the simulation, which may reduce its performance effectiveness. Figure 9 and Figure 10 show a clustered column and a funnel chart of the model’s performance showing how the data followed a linear pattern, with a scale of R^2^ from 0 to 1 for both the training and testing phases. For R^2^, the training result for ANN was 84% while the result for MLR was 97% accuracy, and for the testing result, the R^2^ for ANN was 0.94%, while the R^2^ for MLR was 0.97% accuracy.

## 4. Conclusions

In order to predict COVID-19 cases, this study investigated two data-driven models, one based on artificial neural networks (ANN) and the other using traditional linear regression (MLR). Input parameters were selected from a set of potentially relevant variables. The results demonstrated the MLR and ANN models’ potential as useful instruments for the prediction of COVID-19 cases. Additional models, such as ensemble models, optimization models, and regression models, could be used to improve this study and enhance the performance of the models.

## Figures and Tables

**Table 1:** Results of the Models.

Training				
Models	R^2^	R	RMSE	MSE
ANN	0.846	0.919	0.035	0.001
MLR	0.971	0.985	0.037	0.001
**Testing**				
ANN	0.943	0.971	0.056	0.003
MLR	0.976	0.988	0.057	0.004

## Data Availability

Data is collected from an open source (kaggle.com) and also available upon the request from the authors.
